# Impact of defensive team variables on goals conceded in the first division of the Spanish soccer league: a 10-year study

**DOI:** 10.5114/biolsport.2025.145914

**Published:** 2025-01-14

**Authors:** Pablo Prieto-González, Víctor Martín, Alejandro Sal-de-Rellán

**Affiliations:** 1 Sport Sciences and Diagnostics Research Group, GSD-HPE Department, Prince Sultan University, Riyadh, Saudi Arabia; 2 Faculty of Health Sciences, Universidad Isabel I de Castilla, Burgos, Spain; 3 Faculty of Social Sciences and Communication, Universidad Europea de Madrid, Madrid, Spain

**Keywords:** Defensive variables, Goals conceded, LaLiga, Soccer performance, Ball possession

## Abstract

This study aimed to analyse the influence of defensive team variables on goals conceded in the first division of the Spanish soccer league (LaLiga) between the 2013–2014 and 2022–2023 seasons. Defensive variables from LaLiga’s first division across ten seasons (2013–2014 to 2022–2023) were analysed, including all teams that participated during this period. Thirty-three defensive metrics were selected from the Wyscout database, focusing on team performance. The selection process was conducted by three independent researchers. Moderate positive correlations were found between goals conceded and opponents’ ball touches (r = 0.410), opponent ball possession (r = 0.358), and opponents’ ball touches in various field areas (0.304 ≤ r ≤ 0.381). Defensive errors also correlated positively, while unsuccessful attempts to challenge a dribbling opponent showed a weak negative correlation. Regression analysis identified that opponents’ ball touches and shots conceded were the most significant factors, explaining 25.5% of the variance in goals conceded. The findings suggest that limiting opponents’ touches, possession, and shots is essential in minimizing goals conceded, as greater opponent control correlates with increased scoring chances. Defensive lapses undermine overall performance, while certain set-piece and defensive actions showed a limited effect. Developing strategies that reduce opponent control and improve challenge success rates is crucial. Further research is recommended across different leagues and categories to enhance understanding of defensive tactics in soccer.

## INTRODUCTION

Soccer is a complex sport characterized by constant and dynamic interactions between the members of two teams [1]. Soccer players must structure their behaviour based on strategic plans established before the match, including specific defensive tactics such as high pressing, low-block formations, and man-to-man marking. These tactics aim to reduce the opponent’s chances by either disrupting their buildup play early or maintaining compactness in the defensive third. During the game, players must also adjust their tactics to meet the immediate demands of an ever-changing opponent [2] In this sense, the analysis of teams’ technical performance and playing style has grown exponentially in recent years [3]. Thanks to technological advances, researchers and sports professionals can access extensive data sets on player and team performance, which increasingly provide accurate and representative performance parameters [4].

Despite this growth in performance analysis, much of the existing research has predominantly focused on offensive aspects, with relatively few studies evaluating the defensive side of the game. Soccer’s defensive tactics are uniquely shaped by the sport’s larger field dimensions and the continuous flow of play, which make controlling space and intercepting attacks particularly challenging [5]. Unlike sports such as basketball or handball, where play is more contained, soccer demands defensive strategies that adapt dynamically to the shifting position of the ball and players. Early studies assessed national teams’ defensive behaviour by recording basic indicators such as goals scored, goals conceded, and points per game [6]. More recent research has expanded on this by examining specific aspects of defensive play, such as ball recovery, including the time taken to regain possession [7], and the location of these recoveries [8] further demonstrated that recovering the ball in central areas of the field not only enhances the chances of launching an effective counterattack but also reduces the opponent’s opportunities to create highquality scoring chances, thereby contributing to a lower number of goals conceded and underscoring the importance of defending these zones.

Another critical defensive element is defensive transitions. Defensive transitions refer to the time-window when a team loses ball possession, but is not yet into its ideal defensive formation [9]. This phase is crucial, as it often requires players to recover their positions rapidly and organize defensive formations to prevent the opposing team from exploiting newly vulnerable spaces. Several studies have emphasized the importance of this phase of play, given the high frequency of goals and dangerous situations that can arise during these moments [10, 11]. In this context, Fernandez-Navarro et al. (2020) [10] quantified defensive pressure by measuring the distance between the ball carrier and the nearest defender. Numerous studies have examined different phases of the game and the defensive behaviour of various playing systems, such as the 1-4-4-2 and 1-5-3-2 formations. The 1-5-3-2 system, for example, has been identified as a more conservative defensive structure, characterized by shorter distances between lines and an emphasis on compactness. These formations aim to minimize space for opponents, yet they may also restrict offensive transitions [12]. Analysing these systems is crucial for understanding how teams manage the delicate balance between solid defence and fluid attack.

Teams commonly assess their defensive performance through a range of metrics, including tackles, defensive errors, shots conceded, and successful clearances [13–15]. However, these metrics alone do not fully capture the complexity of modern defensive play, which now involves coordinated collective efforts and effective transitions. While traditional metrics measure isolated defensive actions, dynamic variables such as duels won, interceptions, and pressures reflect a player’s engagement and ability to disrupt the opponent’s play across different phases of the game. Combining these variables with traditional metrics allows for a more nuanced understanding of defensive effectiveness [16]. This multifaceted approach is essential for identifying the key contributors to defensive solidity, such as controlling central areas and maintaining a high defensive line, which are often linked to fewer goals conceded. For coaches and analysts, this approach allows for the development of more comprehensive defensive strategies, fine-tuning tactics to minimize risk while improving overall team performance [17].

In the context of LaLiga, such insights are particularly significant. The Spanish league is known for its technical style of play, characterized by high possession rates and intricate passing sequences, which demand exceptional defensive organization to neutralize [18]. Understanding how LaLiga teams leverage defensive formations and metrics to minimize goals conceded offers valuable lessons not only for LaLiga but also for teams competing in different environments. Despite LaLiga’s prestige and competitive level [18], much of the existing literature on defensive strategies has focused on other leagues, particularly the English Premier League. This research gap underscores the need for further scientific inquiry into the defensive aspects of LaLiga. By addressing this gap, a deeper understanding of the league’s defensive dynamics can be achieved, enriching broader soccer analytics and providing actionable insights for teams seeking to optimize their defensive strategies.

This study sought to address the existing gap by analysing the influence of various defensive variables on goals conceded in LaLiga’s first division between the 2013–2014 and 2022–2023 seasons. Prior research demonstrated that metrics such as defensive duels, interceptions, and goalkeeper saves were critical in shaping defensive performance and reducing goals conceded. Therefore, the investigation of these key factors aimed to deepen the understanding of what constituted defensive success in soccer. Based on these insights [14], it was hypothesized that these defensive variables significantly influence a team’s defensive effectiveness.

## MATERIALS AND METHODS

### Study design

A nomothetic multidimensional study was conducted, adhering to the ethical principles of the Declaration of Helsinki. The Institutional Review Board at Prince Sultan University, Saudi Arabia (PSU IRB-2024-07-5324) approved the study, ensuring its integrity. The study was conducted in accordance with the STROBE (Strengthening the Reporting of Observational Studies in Epidemiology) checklist.

### Setting

The study utilized data from the first division of the Spanish Soccer League (LaLiga) over 10 seasons, from 2013–2014 to 2022–2023, encompassing 38 league matches per season. The dataset included all 20 teams in the first division each season, totalling 3800 matches. Due to the league’s relegation and promotion system, 32 different teams were analysed over the 10 years. Data were sourced from Wyscout’s statistical database, which aggregates and provides numerical performance metrics from official match events, ensuring standardized and comprehensive data across all matches.

### Teams included in the analysis

To ensure a comprehensive analysis of professional-level data, this cross-sectional study’s eligibility criteria included all professional soccer teams participating in the first division of LaLiga during the specified seasons.

### Variables

Study variables were obtained from the Wyscout database, a widely used and reliable source in sports research that offers comprehensive data on both offensive and defensive metrics for soccer matches [19–21]. This platform has been extensively utilized in previous studies, demonstrating its reliability for evaluating player performance and soccer analytics [21]. It has also proven valuable for match event analysis and player position prediction [19, 20]. In the present study, 33 defensive variables were selected from the 115 available variables in the Wyscout dataset (see Table 1 and Supplementary Material 1). Definitions for these variables were established through consensus among three principal researchers, each holding a Ph.D. in Sports Science with a specialization in soccer. They possess significant research experience in soccer and extensive expertise as strength and conditioning specialists for elite-level soccer teams, and hold a UEFA C-level soccer coaching certification. Only those defensive variables unanimously agreed upon by the researchers were included.

**TABLE 1:** Descriptive statistics of variables included in the study: average per season over the 10 seasons (2013–2014 to 2022–2023) in the First Division of La Liga (38 Match days per Season).

Variable	X ± SD	IC 95% Lower	IC 95% Upper
Goals conceded	47.84 ± 13.02	45.25	50.42
Opponent ball possession	50.29 ± 6.58	48.98	51.59
Opponent goal assists	32.18 ± 9.19	30.35	34.01
Yellow cards	95.02 ± 15.65	91.91	98.12
Red cards	4.71 ± 2.84	4.14	5.27
Shots conceded	149.59 ± 27.01	144.23	154.95
Saves	101.90 ± 19.49	98.03	105.77
Clean sheets	8.90 ± 5.80	7.75	10.05
Penalties conceded	5.17 ± 2.42	4.69	5.65
Penalties saved	1.15 ± 1.08	0.94	1.36
Opponent penalties missed	0.33 ± 0.57	0.22	0.44
Free-kick goals conceded	1.02 ± 1.07	0.81	1.23
Corner kick goals conceded	5.10 ± 2.62	4.58	5.62
Average distance of shots taken by opponents	18.09 ± 0.67	17.96	18.23
Free-kick shots received	18.33 ± 5.34	17.27	19.39
Opponent completed passes	11168.07 ± 5807.52	10015.73	12320.41
Opponent attempted passes	14382.32 ± 7403.84	12913.24	15851.41
Players tackled	587.54 ± 58.94	575.84	599.24
Successful tackles	351.90 ± 42.47	343.47	360.33
Defensive third tackles	286.71 ± 43.44	278.09	295.33
Midfield third challenges	228.10 ± 29.82	212.18	234.02
Attacking third challenges	72.73 ± 14.73	69.81	75.65
Unsuccessful attempts to challenge a dribbling opponent	347.80 ± 44.86	338.90	356.70
Shots blocked	102.45 ± 20.56	98.37	106.53
Ball interceptions	367.40 ± 48.88	357.70	377.10
Clearances	730.57 ± 118.71	707.02	754.12
Defensive errors leading to opponent shots	11.51 ± 4.23	10.67	12.35
Opponents’ ball touches	21965. 87 ± 3325.56	21306.05	22625.69
Opponents’ ball touches inside own penalty area	2224.59 ± 246.27	2175.73	2273.45
Opponents’ ball touches in defensive third	6877.57 ± 836.08	6711.67	7043.47
Opponents’ ball touches in midfield third	9952.62 ± 1766.76	9602.06	10303.18
Opponents’ ball touches in attacking third	5349.66 ± 1232.14	5105.18	5594.14
Opponents’ ball touches inside opponent penalty area	734.37 ± 169.63	700.71	768.03

Legend: X : mean; SD: standard deviation; min: minimum; max: maximum; IC 95% Lower: Lower bound of the 95%. Confidence Interval; IC 95% Upper: Upper bound of the 95% Confidence Interval

### Bias

To address potential sources of bias in our analysis, we assessed the internal consistency of the 33 defensive variables across the 10 seasons using Cronbach’s alpha. This statistical measure evaluates the degree to which items within a set (in this case, our selected variables) consistently reflect the same underlying construct. We obtained Cronbach’s alpha values ranging from 0.872 to 0.971, indicating a high level of internal consistency.

High internal consistency is important as it indicates that the variables are reliably measuring the same concepts over time, which helps to reduce the likelihood of measurement error, which could affect the results. Ensuring that these defensive metrics consistently show stable relationships across seasons contributes to the credibility of the findings and minimizes bias associated with temporal fluctuations or inconsistencies in data collection. This methodical approach provides confidence that the variables used in the study accurately reflect the defensive performance of teams during the specified period, thereby enhancing the overall validity of the results.

### Statistical analyses

Data were summarized as mean ± standard deviation (SD). The normality of data and residuals was evaluated using the Kolmogorov-Smirnov test, while the homoscedasticity of variances was assessed with the Levene test. Pearson’s bivariate correlation coefficient was employed to examine relationships between variables, with interpretation thresholds set as follows: 0 ≤ r ≤ 0.09 (very weak), 0.10 ≤ r ≤ .29 (weak), 0.30 ≤ r ≤ 0.49 (moderate), 0.50 ≤ r ≤ 0.69 (strong), and r ≥ 0.70 (very strong) [22]. Regression analysis was performed to analyse the association between goals conceded (dependent variable) and selected defensive team variables (predictor variables). Scatter plots were used to assess whether a linear or non-linear regression model was appropriate, with multiple linear regression determined to be the best fit. The Durbin-Watson statistic was employed to test the independence of residuals, with values between 1.5 and 2.5 considered acceptable [23]. The stepwise method was chosen for regression analysis due to the large number of predictor variables, ensuring model efficiency and reducing redundancy [24]. Data analysis was performed using IBM SPSS Statistics (Version 26) software.

## RESULTS

The normality of all variables assessment showed *p*-values ranging from 0.060 to 0.200, indicating that the data for all variables met the assumption of normality. Levene’s test for homogeneity of variances revealed *p*-values ranging from 0.096 to 0.531 for the dependent variable, suggesting that the assumption of equal variances was not violated. Additionally, the Durbin-Watson statistic ranged between 1.5 and 2.5, indicating that the assumption of independence of residuals was satisfied. Additionally, the variance inflation factor values were below 10, and the collinearity tolerance values exceeded 0.1. These results confirmed that the basic assumptions for conducting multiple regression analysis were satisfied. Afterward, Pearson’s bivariate correlation results (see Table 2 and Supplementary Material 2) showed that the following variables exhibited a moderate, positive, and significant correlation with goals conceded in this sequence: opponents’ ball touches (r = 0.410, *p* < 0.0001), opponents’ ball touches inside the opponent penalty area (r = 0.381, *p* < 0.0001), opponents’ ball touches in the midfield third (r = 0.371, *p* < 0.0001), opponent ball possession (%) (r = 0.358, *p* < 0.0001), opponents’ ball touches in the defensive third (r = 0.353, *p* < 0.0001), opponents’ ball touches in the attacking third (r = 0.336, *p* = 0.001), and opponents’ ball touches inside their own penalty area (r = 0.304, *p* = 0.002). Furthermore, defensive errors leading to opponent shots showed a weak, positive, and significant correlation with goals conceded. In contrast, unsuccessful attempts to challenge a dribbling opponent showed a weak, negative, and significant correlation with goals conceded.

**TABLE 2:** Correlation between the team offensive variables selected and the goals conceded

Variable	r	p
Opponents’ ball touches	.410*	< .0001
Opponents’ ball touches inside opponent penalty area	.381*	< .0001
Opponents’ ball touches in midfield third	.371*	< .0001
Opponent ball possession (%)	.358*	< .0001
Opponents’ ball touches in defensive third	.353*	< .0001
Opponents’ ball touches in attacking third	.336*	.001
Opponents’ ball touches inside own penalty area	.304*	.002
Defensive errors leading to opponent shots	.264*	.008
Unsuccessful attempts to challenge a dribbling opponent	-.211*	.035
Attacking third challenges	.114	.259
Shots blocked	.114	.258
Free-kick goals conceded	.109	.282
Saves	.096	.341
Shots conceded	.081	.426
Penalties saved	.048	.636
Opponent penalties missed	.030	.709
Clean sheets	.020	.840
Successful tackles	.020	.846
Opponent goal assists	.004	.965
Defensive third tackles	.001	.994
Midfield third challenges	.001	.992
Corner kick goals conceded	-.013	.899
Penalties conceded	-.019	.853
Average distance of shots taken by opponents	-.022	.827
Opponent penalties missed	-.038	.709
Free-kick shots received	-.050	.624
Red cards	-.068	.503
Opponent completed passes	-.117	.244
Clearances	-.118	.248
Opponent attempted passes	-.125	.217
Ball interceptions	-.125	.214
Yellow cards	-.131	.193

Legend: r: Coefficient of Correlation; p: Significance Level:

* :significant correlation found; n = 200

In the regression analysis, goals conceded was set as the dependent variable, and the remaining team defensive variables were used as the independent variables, generating two models (see Table 3). In Model 1, the variable included was opponents’ ball touches, while in Model 2, the variables included were opponents’ ball touches and shots conceded, ordered by importance.

**TABLE 3:** Stepwise multilinear regression analysis of the association between goals conceded and team offensive variables.

Analysis	R2	*p*-Value (model)	Dependent Variable	Independent Variables	Standardized Coecient (β)	*p*-Value (variable)
Model 1	.168	< 0.001	Goals conceded	Opponents’ ball touches	0.410	< 0.001
Model 2	.255	< 0.001	Goals conceded	Opponents’ ball touches	.556	< 0.001
				Shots conceded	.328	< 0.001

Legend: R^2^: Coefficient of Determination; *p*-Value (model): Significance Level of the Model; Standardized Coefficient (β): Standardized coefficient beta; *p*-Value (variable): Significance Level of each independent variable

## DISCUSSION

This study aimed to determine the influence of teams’ defensive variables on the goals conceded in the first division of the Spanish soccer league between the 2013–2014 and 2022–2023 seasons. Through the analysis of these variables, we have deepened the understanding of defensive success in soccer. The main findings indicated that the following variables presented a significant positive correlation, in decreasing order from moderate to weak: opponents’ ball touches, opponents’ ball touches inside the penalty area, opponents’ ball touches in the midfield third, opponent ball possession, opponents’ ball touches in the defensive third, opponents’ ball touches in the attacking third, opponents’ ball touches inside the team’s own penalty area, and defensive errors leading to opponent shots. Additionally, unsuccessful attempts to challenge a dribbling opponent showed a significant, weak negative correlation with goals conceded. Furthermore, according to the multiple regression analysis conducted, the variables of opponents’ ball touches and shots conceded are explanatory of the goals conceded.

The current findings identified that opponents’ ball touches presented the highest correlation with goals conceded among the analysed variables. This result may reflect that a greater number of touches by the opposing team indicates more control of the game and, therefore, more opportunities to create attacking plays that can culminate in goals. Previous studies obtained results similar to ours, showing that successful teams perform a higher number of ball touches [25]. Additionally, teams with a high percentage of ball possession exhibited superior technical quality indicators, such as a higher number of effective touches and passes [26]. Indeed, it has been highlighted that players’ technical skills are crucial for increasing ball possession [27, 28], and that longer durations of ball possession imply a higher number of touches [29]. Furthermore, as regards the ball touches, a positive relationship was observed between opponents’ ball touches in the defensive third and goals conceded. This association may be due to the fact that more successful teams initiate play from their own area with short passes. However, defensive strategies, such as high pressing or compact formations, may also influence this relationship. For example, teams employing high pressing disrupt opponents’ build-up play, potentially reducing 1 v 1 dribbling challenges and limiting opportunities to concede. In contrast, compact defensive structures aim to restrict space, forcing opponents to play laterally or backward, which reduces goal-scoring chances when individual challenges are unsuccessful. LaLiga’s playing style, emphasizing possession and short, controlled passing, likely impacts these patterns. Top teams, such as Barcelona and Real Madrid, often maintain high possession, building attacks from the back with precision. This possession-based style, common in LaLiga [28], may increase touches in the defensive third among successful teams, thus shaping the observed relationship between ball touches and goals conceded. Yi et al. (2019) recorded that during the FIFA World Cup (FWC) 2018, the passing performance and goals scored by possession teams were higher than those of direct play teams [30]. Moreover, in the FWC 2010, teams with more possession had more opportunities to score [31]. However, Sarmento et al. (2018) reported that the offensive efficiency of counterattacks was 40% higher than that of positional attacks [3]. These results should be interpreted with caution, as the success and style of play vary significantly across leagues, reflecting their unique tactical philosophies. For example, the English Premier League is often associated with a more direct style of play, characterized by fast-paced transitions and a greater emphasis on counterattacks. In contrast, LaLiga teams typically prioritize ball possession and intricate passing sequences [32]. This difference can influence gameplay strategies and ultimately impact team performance. These contrasting styles illustrate how league characteristics shape not only tactical decisions but also the effectiveness of teams in achieving success.

Our results showed that defensive errors leading to opponent shots also correlated with goals conceded. In line with this, previous studies have demonstrated that winning teams make fewer defensive errors [14]. Moreover, losing the ball near one’s own goal is more dangerous [10]. Specifically, after losses in the defensive zone, there are seven times more goals and nineteen times more shots in the goal area [30], compared to losses in the offensive zone. Additionally, 3.64% of the goals in the 2010 World Cup were attributed to defensive errors, highlighting the direct impact of such errors on goals conceded in high-level tournaments [33]. Finally, unsuccessful attempts to deny a dribbling opponent could be related to goals conceded, allowing the attacker to advance and create goal-scoring situations. Previous studies have shown that breaking the defensive line with a dribble offers a tactical advantage and may therefore increase the chance of scoring a goal [15, 30].

Furthermore, the reasons why unsuccessful attempts to challenge a dribbling opponent negatively correlated with goals conceded could be due to the defensive team’s ability to compensate strategically in 1 v 1 situations. Unsuccessful challenges may not necessarily result in direct goal-scoring opportunities if the defensive team’s structure is strong enough to absorb the loss from these encounters. A well-organized defensive setup can mitigate threats even when individual challenges fail. Defenders may rely on positioning, support from teammates, and tactical adjustments to close down space effectively, which can prevent goal-scoring despite unsuccessful challenges. Further analysis of defensive cohesion and the tactical response to 1 v 1 situations could provide additional clarity on this relationship. This finding aligns with previous research that underscores the significance of team-based defensive strategies in minimizing the impact of individual errors [34].

According to our regression analysis, aside from the variable of opponents’ ball touches, shots conceded showed a significant predictive capability for goals conceded. This outcome aligns with the intuitive assumption that a higher number of shots taken by opponents increases the likelihood of them scoring goals. Previous studies in LaLiga have similarly demonstrated that successful teams are those with both high shooting accuracy and a reduced number of shots allowed to opponents, highlighting the importance of efficient defensive strategies [35, 36]. Similar findings have been reported in both national and international competitions, reinforcing the value of minimizing conceded shots to improve defensive outcomes. [37, 38]. However, in contrast, a recent study by Gonzalez-Rodenas et al. (2023) reported no significant correlation between shots on goal and actual goals scored, suggesting that while this relationship is foundational in soccer, there are complex variables at play that might influence its consistency [39]. Although there is some controversy in these results, this relationship is fundamental in soccer and highlights the importance of having strong defensive strategies in place to limit the number of shots that can be taken by the opponent. In relation with these findings, our results indicated no direct relationship between the average distance of opponents’ shots and goals conceded, which may be attributed to shot quality. Teams with high shot accuracy can score even from longer distances, a factor emphasized in prior research [40]. In contrast, teams with lower shot accuracy tend to be less effective with long-range shots, reducing the threat they pose from these distances [36].

Another influential factor is goalkeeper performance, as the success of shots – especially those from longer ranges – depends significantly on the goalkeeper’s ability to make saves. Studies have shown that goalkeepers with excellent reflexes, positioning, and shot-stopping skills can effectively limit goals conceded from high-quality shots, regardless of shot distance [41].

Furthermore, Rathke [2017] emphasized the importance of both shot distance and angle in expected goal (xG) models, which predict scoring potential [42]. His findings suggest that while distance alone is relevant, the angle of the shot also plays a crucial role in shot efficiency, indicating that a combination of these factors may be more predictive of goal outcomes than distance alone. Integrating shot quality, goalkeeper effectiveness, and shot angle into defensive strategies could therefore enhance the understanding and management of factors affecting goal concession.

As for the defensive variables that did not correlate with goals conceded, set-piece metrics – free-kick goals conceded, penalties saved, missed opponent penalties, corner-kick goals conceded, and free-kick shots received – showed no significant correlation with overall goals conceded. This could be partly due to their relatively low frequency over a season, which may limit their overall impact. Indeed, as shown in Table 1, free-kick goals conceded account for only 2.13% of total goals, while penalty goals conceded make up 7.21%. Additionally, factors such as defensive organization during set pieces, individual marking assignments, and situational variables (i.e., weather conditions or luck) may further weaken the relationship between setpiece opportunities and goals conceded [43]. Defensive strategies specifically designed for set-piece situations – such as zone marking and mixed defensive systems – can also diminish the effectiveness of opponents’ set pieces, further reducing their impact on total goals conceded [44]. Another variable that did not correlate with goals conceded is the disciplinary aspect (i.e., red and yellow cards). It seems that the lack of correlation in the case of red cards is due to the fact that, although sending-offs can influence the outcome of the match, coaches often try to counteract the disadvantage with defensive tactical modifications [45]. Contrary to our findings, Bar-Eli et al. (2006) concluded that the chances of a penalized team scoring goals or winning were substantially reduced after the penalty [46]. Despite this, although cards do not directly correlate with goals, they may have other negative effects on performance. Lago-Peñas et al. (2016) found that playing 11 vs. 10 increases time of possession, the number of total passes, short passes, total touches, and the percentage of successful passes compared to playing 11 vs. 11 [32]. In addition, the same study revealed that teams playing with a numerical advantage spent less time defending, while the penalized team performed worse in all variables after the sending-off.

Regarding yellow cards, previous studies [47, 48] indicated that they have a negative effect on the goal percentage of the cautioned team. Furthermore, another study showed that teams with a higher number of cautioned players have a lower goal-scoring rate when they are winning [49]. The opponent’s completed passes and attempted passes did not correlate with goals conceded. This result may be because high values in these parameters do not necessarily reflect the risk taken in those passes, their difficulty, or the areas of the field where they are made [32, 33]. To enable an objective interpretation of the results discussed above, the main limitations of this study should be noted. First, all the variables evaluated in this research are related to the defensive process, regardless of tactical formation or situational variables, which can influence the outcome. Furthermore, this investigation was performed with data from LaLiga (Spain) involving professional male players, and the results should not be extrapolated to other leagues. Moreover, it could be very interesting to replicate these results in other categories or in women’s competitions. However, the large sample of teams analysed in this study reinforces the consistency of the findings, which have important practical applications.

## CONCLUSIONS

This study highlights the importance of minimizing ball touches, opponent possession, and shots received to reduce goals conceded, emphasizing that defensive success results from the complex interplay of multiple interrelated factors rather than relying on isolated metrics. Greater control of the ball by the opponent translates to more scoring opportunities, while defensive errors that lead to shots have a negative impact on defensive performance. Although certain defensive actions, such as set pieces and disciplinary sanctions, do not show a direct correlation with goals conceded, they do influence the overall performance of the team.

To address the complexity observed in defensive success, coaches may consider optimizing various defensive actions according to game context. For instance, a combination of midfield pressure along with training focused on reducing defensive errors could be more effective in high-risk situations, helping to prevent opponents from advancing into scoring areas. Teams can prioritize these strategically combined actions tailored to match circumstances to limit the opponent’s scoring opportunities. Additionally, these findings suggest a need for comprehensive defensive strategies that integrate multiple actions and tactical decisions, alongside continuous evaluation of defensive dynamics across leagues and player categories. As soccer evolves, a detailed understanding of how these factors interact in different game scenarios can offer coaches and analysts a more precise guide to improving the defensive capabilities of their teams.

### Practical applications

To enhance defensive effectiveness, teams should prioritize minimizing opponents’ ball touches, possession, and shots conceded. Training programmes should focus on defensive organization and situational drills that prepare players to regain possession under pressure. Coaches need to implement tactical formations that promote compactness and disrupt the opponent’s control of the game. Additionally, teams must emphasize communication among defenders to reduce errors that can lead to scoring chances. While disciplinary actions and set-piece metrics might not directly affect goals conceded, maintaining a disciplined approach can positively influence overall performance.

## Supplementary Material

Impact of defensive team variables on goals conceded in the first division of the Spanish soccer league: a 10-year study
