# Supplementary material for: Impact of defensive team variables on goals conceded in the first division of the Spanish soccer league: a 10-year study
Source: Biol Sport. 2025 Jan 14;42(3):17–28. doi: 10.5114/biolsport.2025.145914 (PMC12244413; doi:10.5114/biolsport.2025.145914)
Supplement: Impact of defensive team variables on goals conceded in the first division of the Spanish soccer league: a 10-year study [file JBS-42-3-55338-s1.pdf]

SUPPLEMENTARY MATERIAL 1. DEFINITIONS OF KEY DEFENSIVE VARIABLES SELECTED

- **Attacking Third Challenges**: The number of challenges or duels made by a team in the opponent's attacking third of the field, reflecting defensive pressure in high-risk zones.
- **Average Distance of Shots Taken by Opponents**: The mean distance from the goal at which opposing teams take their shots, providing insight into how well the defense restricts opponents' shooting opportunities to outside the penalty area.
- **Ball Interceptions**: When a player anticipates and intercepts the ball during an opponent's pass or play, disrupting their attack without the need for a tackle.
- **Clean Sheets**: A match in which a team prevents the opponent from scoring any goals.
- **Clearances**: Defensive actions where the ball is kicked or headed away from the defensive zone, typically to remove immediate pressure from an attacking threat.
- **Corner Kick Goals Conceded**: The number of goals a team allows from corner kicks, reflecting defensive vulnerabilities in set-piece situations.
- **Defensive Errors Leading to Opponent Shots**: Mistakes made by defenders that directly result in the opposition taking a shot, an indicator of lapses in defensive concentration or poor decision-making.
- **Defensive Third Tackles**: Tackles made by a team in its own defensive third, used to stop an opponent's attack and regain possession.
- **Free-Kick Goals Conceded**: The number of goals a team concedes directly from free-kicks, reflecting defensive and goalkeeper weaknesses in defending set-pieces.
- **Free-Kick Shots Received**: The total number of shots an opponent attempts directly from free-kicks, measuring the frequency of fouls committed in dangerous areas.
- **Goals Conceded**: The total number of goals allowed by a team over the course of a match or season.
- **Midfield Third Challenges**: The number of defensive challenges or duels a team engages in within the midfield third, highlighting the team's defensive activity in preventing opposition progression through the middle of the field.
- **Opponent Attempted Passes**: The number of passes attempted by the opposing team during a match.
- **Opponent Ball Possession**: The percentage of time the opponent controls the ball during the game, indicating the amount of defensive work a team has to perform.
- **Opponent Completed Passes**: The number of passes completed by the opposition during the game, used to measure how well the defending team disrupts passing lanes.
- **Opponent Goal Assists**: The number of assists made by opponents, which contribute to the goals they score against the defending team.
- **Opponent Penalties Missed**: The total number of penalty kicks missed by the opposition, reflecting either defensive discipline or the goalkeeper's ability to prevent goals from penalty situations.
- **Opponents' Ball Touches**: The total number of times the opponent touches the ball during a match, which provides insight into how much ball control the defending team allows.
- **Opponents' Ball Touches in Attacking Third**: The number of ball touches by the opponent in the attacking third, indicating the frequency with which the opposition enters dangerous attacking positions.
- **Opponents' Ball Touches in Defensive Third**: The number of touches the opposing team makes in the defending team's defensive third, indicating how much pressure they are under in their own zone.
- **Opponents' Ball Touches in Midfield Third**: The number of ball touches made by the opponent in the midfield third of the pitch, reflecting their control in the central areas.
- **Opponents' Ball Touches Inside Opponent Penalty Area**: The number of ball touches the opponent makes inside their own penalty area, often indicating a team's defensive pressure that forces the opponent back.
- **Opponents' Ball Touches Inside Own Penalty Area**: The number of touches the opposing team makes inside the defending team's penalty area, highlighting how frequently the defense is breached and exposed to goal-scoring threats.
- **Penalties Conceded**: The number of penalty kicks awarded to the opponent as a result of fouls or handballs inside the defending team's penalty area.
- **Penalties Saved**: The number of penalty kicks successfully saved by the goalkeeper, preventing the opposition from scoring.
- **Players Tackled**: The number of opposing players successfully tackled during a game, resulting in the defending team regaining possession.
- **Red Cards**: The number of times players receive a red card, resulting in their immediate ejection from the game and a reduction in team size for the remainder of the match.
- **Saves**: The number of shots on target stopped by the goalkeeper, preventing goals.
- **Shots Blocked**: The number of opponent shots blocked by defenders before the ball reaches the goalkeeper, preventing goal-scoring opportunities.
- **Shots Conceded**: The total number of shots the defending team allows the opposition to take during the match, an indicator of defensive pressure.
- **Successful Tackles**: The number of tackles in which the defender successfully takes the ball away from the opponent without committing a foul.
- **Unsuccessful Attempts to Challenge a Dribbling Opponent**: The number of failed attempts to dispossess an opponent who is dribbling the ball, resulting in the opponent retaining possession.
- **Yellow Cards**: The number of times players receive a yellow card, indicating caution for infractions that, if repeated, could lead to a red card.

SUPPLEMENTARY MATERIAL 2. CORRELATION MATRIX OF ALL TEAM DEFENSIVE VARIABLES

	GC	OBP	OGA	YC	RC	SC	SV	CS	PC	PS	OPM	FKG	CKG	ASDO	FKS	OCP
OBP	-.358**															
OGA	0.004	.419**														
YC	-0.131	.455**	.192*													
RC	-0.068	.193*	.232*	.394**												
SC	0.081	.439**	.697**	.189*	.246**											
SV	0.096	.316**	.403**	0.12	.173*	.922**										
CS	0.02	-.222*	-.470**	-0.13	-0.032	-.509**	-.394**									
PC	-0.019	.335**	.303**	.181*	0.097	.403**	.261**	-.217*								
PS	0.048	.252**	.226*	0.091	0.021	.280**	0.159	-0.004	.176*							
OPM	-0.038	0.004	0.039	-0.131	0.029	0.009	0.039	-0.036	-.269**	-0.082						
FKG	0.109	0.032	0.063	0.021	-0.104	.165*	0.109	-.287**	.205*	-0.038	-0.077					
CKG	-0.013	.204*	.415**	-0.006	0.084	.360**	.265**	-.198*	0.095	0.159	-0.016	-0.123				
ASDO	-0.022	-0.065	-.434**	0.132	-0.119	-.281**	-0.152	.252**	0.036	0.001	-.234**	0.107	-0.124			
FKS	-0.05	.282**	0.152	.227*	0.078	.343**	.341**	-.291**	.306**	0.072	-0.132	.360**	0.053	.243**		
OCP	-0.117	.199*	0.087	-0.041	.187*	-0.047	-0.099	.686**	0.048	0.148	0.114	-.256**	0.039	0.053	-0.107	
OAP	-0.125	.187*	0.078	-0.038	.177*	-0.071	-0.126	.702**	0.036	0.144	0.116	-.258**	0.027	0.044	-0.119	.998**
PT	0.03	0.083	0.042	.317**	0.106	0.072	0.084	-.281**	0.02	-0.047	-0.029	0.044	0.051	-0.018	0.088	-.369**
ST	0.02	0.009	-0.038	.298**	0.128	0.072	0.128	-.329**	-0.009	-0.09	-0.033	0.043	0.006	0.003	0.12	-.491**
DTT	0.001	.273**	.278**	.279**	.185*	.430**	.414**	-.383**	.235**	0.027	-0.023	0.129	.201*	-0.062	.334**	-.229*
MTC	0.001	0	-0.142	.276**	-0.003	-.270**	-.269**	-0.08	-0.076	-0.055	-0.056	-0.009	-0.089	0.05	-0.103	-.294**
ATC	0.114	-.471**	-.366**	-0.113	-0.113	-.434**	-.341**	.169*	-.457**	-0.159	0.065	-.188*	-.208*	0.008	-.428**	-.206*
UACDO	-.211*	.347**	0.158	.183*	-0.022	.220*	.174*	-.223*	.285**	0.072	-0.046	0.086	0.118	0.019	.375**	-0.061
SB	0.114	.249**	.238**	0.138	0.151	.613**	.660**	-.167*	.207*	0.165	-0.081	0.161	0.078	0.105	.289**	0.048
BI	-0.125	.320**	0.12	0.049	-0.133	0.014	-0.088	-.404**	.182*	0.041	-.169*	.193*	0.059	-0.012	.297**	-.350**
C	-0.118	.636**	.399**	.354**	0.127	.555**	.496**	-.569**	.257**	0.1	-0.034	.294**	.190*	-0.056	.455**	-.330**
DELOS	.264**	-.375**	-0.103	-0.111	-0.029	0.025	0.107	-.207*	-.210*	-.223*	0.135	.202*	-0.102	-0.11	-0.045	-.340**
TOT	.410**	-.909**	-.492**	-.505**	-.219*	-.446**	-.291**	.259**	-.304**	-.237**	-0.057	-0.069	-.190*	0.152	-.289**	-.177*
OBTPA	.304**	-.225*	.201*	-.317**	-0.012	.429**	.471**	-0.011	0.102	0.079	0.045	0.105	.174*	-0.03	0.12	0.127
OBTD	.353**	-.554**	-0.052	-.420**	-0.116	0.104	.190*	0.075	-0.042	-0.046	0.024	0.068	0.092	0.097	0.041	0.006
OBTTM	.371**	-.885**	-.521**	-.467**	-.220*	-.507**	-.362**	.250**	-.315**	-.236**	-0.069	-0.054	-.216*	.178*	-.282**	-.219*
OBTTA	.336**	-.813**	-.545**	-.408**	-.198*	-.543**	-.393**	.283**	-.338**	-.274**	-0.075	-0.153	-.264**	0.085	-.402**	-.176*
OBTPPA	.381**	-.798**	-.549**	-.367**	-.174*	-.470**	-.297**	.263**	-.302**	-.291**	-0.096	-0.14	-.245**	0.148	-.392**	-.208*

aLegend: GC: Goals conceded; OBP: Opponent ball possession; OGA: Opponent goal assists; YC: Yellow cards; RC: Red cards; SC: Shots conceded; SV: Saves; CS: Clean sheets; PC: Penalties conceded; PS: Penalties saved; OPM: Opponent penalties missed; FKG: Free-kick goals conceded; CKG: Corner kick goals conceded; ASDO: Average distance of shots taken by opponents; FKS: Free-kick shots received; OCP: Opponent completed passes; OAP: Opponent attempted passes; PT: Players tackled; ST: Successful tackles; DTT: Defensive third tackles; MTC: Midfield third challenges; ATC: Attacking third challenges; UACDO: Unsuccessful attempts to challenge a dribbling opponent; SB: Shots blocked; BI: Ball interceptions; C: Clearances; DELOS: Defensive errors leading to opponent shots; TOT: Opponents' ball touches; OBTPA: Opponents' ball touches inside own penalty area; OBTD: Opponents' ball touches in defensive third; OBTTM: Opponents' ball touches in midfield third; OBTTA: Opponents' ball touches in attacking third; OBTPPA: Opponents' ball touches inside opponent penalty area; *: Correlation is significant at the 0.01 level; **: Correlation is significant at the 0.05 level.

SUPPLEMENTARY MATERIAL 2. CONTINUE

	OAP	PT	ST	DTT	MTC	ATC	UACDO	SB	BI	C	DELOS	TOT	OBTPA	OBTD	OBTTM	OBTTA
OBP																
OGA																
YC																
RC																
SC																
SV																
CS																
PC																
PS																
OPM																
FKG																
CKG																
ASDO																
FKS																
OCP																
OAP																
PT	-.376**															
ST	-.500**	.925**														
DTT	-.255**	.759**	.690**													
MTC	-.280**	.751**	.674**	.190*												
ATC	-.186*	.244**	.300**	-.298**	.422**											
UACDO	-0.06	.336**	.274**	.374**	.221*	-.206*										
SB	0.019	0.049	0.022	.301**	-.213*	-.258**	0.018									
BI	-.348**	0.149	0.11	0.165	0.164	-.225*	.445**	-.232*								
C	-.347**	.347**	.315**	.581**	0.058	-.441**	.396**	.457**	.351**							
DELOS	-.339**	0.085	0.113	0.101	-0.032	0.107	-.194*	-0.017	-0.144	-0.061						
TOT	-.173*	-0.106	-0.034	-.282**	-0.013	.438**	-.328**	-.242**	-.278**	-.647**	.328**					
OBTPA	0.1	-0.163	-.192*	.195*	-.469**	-.280**	-0.101	.561**	-.359**	0.09	.222*	.268**				
OBTD	-0.012	-0.115	-0.133	0.128	-.353**	-0.124	-0.11	.259**	-.288**	-.179*	.314**	.605**	.859**			
OBTTM	-.211*	-0.074	-0.01	-.302**	0.069	.456**	-.304**	-.306**	-.211*	-.624**	.306**	.977**	0.15	.498**		
OBTTA	-0.165	-0.097	0.021	-.413**	0.106	.615**	-.372**	-.391**	-.249**	-.728**	.236**	.890**	-0.075	.242**	.865**	
OBTPPA	-.204*	-0.044	0.068	-.352**	0.133	.594**	-.428**	-.248**	-.310**	-.635**	.242**	.853**	-0.003	.297**	.822**	.927**

a*Legend:* GC: Goals conceded; OBP: Opponent ball possession; OGA: Opponent goal assists; YC: Yellow cards; RC: Red cards; SC: Shots conceded; SV: Saves; CS: Clean sheets; PC: Penalties conceded; PS: Penalties saved; OPM: Opponent penalties missed; FKG: Free-kick goals conceded; CKG: Corner kick goals conceded; ASDO: Average distance of shots taken by opponents; FKS: Free-kick shots received; OCP: Opponent completed passes; OAP: Opponent attempted passes; PT: Players tackled; ST: Successful tackles; DTT: Defensive third tackles; MTC: Midfield third challenges; ATC: Attacking third challenges; UACDO: Unsuccessful attempts to challenge a dribbling opponent; SB: Shots blocked; BI: Ball interceptions; C: Clearances; DELOS: Defensive errors leading to opponent shots; TOT: Opponents' ball touches; OBTPA: Opponents' ball touches inside own penalty area; OBTD: Opponents' ball touches in defensive third; OBTTM: Opponents' ball touches in midfield third; OBTTA: Opponents' ball touches in attacking third; OBTPPA: Opponents' ball touches inside opponent penalty area; *: Correlation is significant at the 0.01 level; **: Correlation is significant at the 0.05 level.

Articles published in the Biology of Sport are licensed under an open access Creative Commons CC BY 4.0 license.
